# Veränderungen von Wohlbefinden und privater Unterstützung für Ältere: ein Blick auf die Auswirkungen der COVID-19-Pandemie im Frühsommer 2020

**DOI:** 10.1007/s00391-021-01870-2

**Published:** 2021-03-05

**Authors:** Martina Brandt, Claudius Garten, Miriam Grates, Judith Kaschowitz, Nekehia Quashie, Alina Schmitz

**Affiliations:** Fakultät Sozialwissenschaften, TU Dortmund, Emil-Figge-Str. 50, 44227 Dortmund, Deutschland

**Keywords:** Sorgearbeit, Einsamkeit, Lebenszufriedenheit, Geschlechterungleichheit, Alter, Care work, Loneliness, Life satisfaction, Gender inequality, Age

## Abstract

**Hintergrund:**

Die Pilotstudie „Gesundheit und Unterstützung in Zeiten von Corona“ (Technische Universität Dortmund) erhob vom Mai bis Juli 2020 Veränderungen von Unterstützung und Wohlbefinden Älterer infolge der COVID-19-Pandemie.

**Ziel der Arbeit:**

Ziel war es, empirische Erkenntnisse zu den sozialen und mentalen Folgen der Pandemie für in Privathaushalten lebende Personen der Altersgruppe 40+ Jahre zu gewinnen. Betrachtet wurden durch die Pandemie bedingte Änderungen im Erhalt und im Leisten von Unterstützung (u. a. persönliche Pflege, Hilfe im Haushalt) und Betreuungsprobleme sowie Veränderungen des Wohlbefindens.

**Material und Methoden:**

Mithilfe deskriptiver und multivariater Analysen wurde untersucht, wie sich im Zuge der Pandemie Unterstützungsmuster änderten, ob Betreuungsprobleme entstanden, und ob sich in diesem Zuge des Wohlbefinden (Lebenszufriedenheit und Einsamkeit) veränderte.

**Ergebnisse:**

Im Zuge der Pandemie zogen sich insbesondere Ältere und Hochaltrige aus der Unterstützung für andere zurück. Frauen berichteten häufiger von Betreuungsproblemen mit älteren Angehörigen. Das Wohlbefinden verringerte sich insgesamt, am deutlichsten aber bei Frauen und Hochaltrigen. Die multivariaten Analysen verdeutlichen, dass Betreuungsprobleme mit älteren Angehörigen im Zuge der Pandemie mit geringerem Wohlbefinden einhergingen.

**Diskussion:**

Unsere Pilotstudie zeigt deutliche Änderungen in Unterstützungsmustern und im Wohlbefinden der Befragten. Viele berichten von mehr Einsamkeit und geringerer Lebenszufriedenheit als vor der Pandemie – insbesondere Frauen, die Unterstützungsleistungen für andere erbringen. Sorgearbeit wird durch die Pandemie und die Maßnahmen zu ihrer Bekämpfung erschwert. Zukünftige Kontaktbeschränkungen sollten mit Bedacht eingesetzt werden und dies im Blick haben.

## Abstract (en)

**Background:**

The pilot study “Health and Support in Times of Corona” (TU Dortmund University) collected data on support and well-being of individuals aged 40 plus years in the light of the first wave of the COVID-19 pandemic from May to July 2020.

**Objective:**

The aim was to study the social and mental effects of the pandemic. We focused on individuals living in private households aged 40 years and older. Participants were asked about pandemic-related changes in receiving and providing support (e.g. personal care, help with household chores), problems arising in taking care of older persons and changes in well-being.

**Material and methods:**

We conducted descriptive and multivariate analyses to show how support changed, problems with support came up and well-being changed in the light of the pandemic and how all this was related.

**Results:**

Due to the pandemic older respondents in particular were no longer able to provide the necessary support for others. Especially women reported problems in taking care of older individuals. We found a decrease in well-being for all respondents but most significantly for women and individuals aged 80 years and older. Moreover, problems in the provision of care due to the pandemic and lower well-being were clearly linked.

**Conclusion:**

Our study showed significant changes in support patterns and well-being due to the COVID-19 pandemic. A substantial part of the respondents reported more loneliness and lower life satisfaction compared to before the pandemic, especially women supporting others. In these pandemic times, informal caregiving is severely hampered. Future pandemic-related measures should be carefully planned bearing such issues in mind.

Die gesundheitlichen Risiken und sozialen wie mentalen Folgen der COVID-19-Pandemie sind ungleich verteilt. Eine Pilotstudie der Technischen Universität (TU) Dortmund untersucht die Veränderungen von Unterstützung und Wohlbefinden Älterer infolge der Pandemie im Frühsommer 2020. Ein verringertes Wohlbefinden ist für Personen zu erwarten, die private Unterstützung leisten (müssen), da Unterstützungsleistungen aufgrund von Maßnahmen zur Eindämmung der Pandemie erschwert werden. Betroffen sind auch Personen, die auf Unterstützung angewiesen sind, diese aber aufgrund der Krise nicht mehr in ausreichendem Maße erhalten.

## Hintergrund

Seit dem Aufkommen der ersten COVID-19-Fälle in Deutschland und den Maßnahmen zur Eindämmung des Virus stellt sich über die gesundheitlichen Risiken hinaus die Frage nach den sozialen und mentalen Folgen der Pandemie [7, 8]. Von zentraler Bedeutung sind die mehrfach eingeführten und angepassten Bestimmungen zur Einschränkung sozialer Kontakte [1]. Diese Maßnahmen erhöhen die Gefahr von Vereinsamung und verringertem Wohlbefinden [7, 9, 18]. Dabei lastet u. a. besonderer Druck auf Personen, die sich um pflegebedürftige Angehörige kümmern – v. a., wenn die Versorgung durch externe Anbieter nicht mehr sichergestellt ist oder zu risikoreich erscheint [2, 9, 16]. Prekär ist die Situation auch für Ältere, die aufgrund ihres höheren Risikos für einen schweren Krankheitsverlauf besonderen Schutz benötigen, aber gleichzeitig wichtiger Partizipationsmöglichkeiten beraubt werden [2, 8]. Durch den Rückzug von professionellen oder privaten HelferInnen erhalten sie ggf. die benötigte Unterstützung nicht mehr [20].

In der vorliegenden Studie wird untersucht, ob sich Wohlbefinden (Lebenszufriedenheit und Einsamkeit) und Unterstützungsmuster bei in Privathaushalten lebenden Personen im mittleren und höheren Erwachsenenalter (40+ Jahre) durch die COVID-19-Pandemie im Frühsommer 2020 verändert haben. Geleistete (private) und erhaltene (private wie professionelle) Unterstützung kann persönliche Pflege, Hilfe im Haushalt, Hilfe bei Schreibarbeiten und sonstige Hilfen umfassen. Die folgenden Analysen beruhen auf Daten einer Pilotstudie der TU Dortmund, die vom Mai bis Juli 2020, gegen Ende der „ersten Welle“, erhoben wurden. In diesem Zeitraum wurden die Kontaktbeschränkungen gerade wieder gelockert, wobei auch im Frühsommer noch Auflagen für Besuche in Pflegeheimen galten [10]. Der Fokus liegt auf Wohlbefinden und Unterstützung Älterer, einem Thema, zu dem es in der frühen Phase der Pandemie kaum empirische Studien gab.

Die Forschungsfragen lauten: Beeinflusste die Pandemie Unterstützungsleistungen und erhielten Personen die Unterstützung, die sie benötigten? Wie änderte sich das subjektive Wohlbefinden, und welche Rolle spielten dabei Schwierigkeiten in der Versorgung älterer Angehöriger? Diese Fragen haben Stand heute – in der zweiten Welle – wieder größte Aktualität und Relevanz auch mit Blick auf künftige Strategien zur Eindämmung der Pandemie.

## Forschungsstand: Unterstützung und Wohlbefinden in der Krise

Soziale Kontakte und gegenseitige Unterstützung innerhalb des sozialen Netzwerks sind eng mit dem subjektiven Wohlbefinden verbunden [4]. Soziale Isolation und das Gefühl von Einsamkeit sind dagegen ein Risiko für die mentale Gesundheit [4]. Bekannt ist, dass Ältere häufiger von Einsamkeit betroffen sind als Personen im mittleren Erwachsenenalter [11]. Das Einsamkeitsrisiko ist zudem erhöht, wenn intensive Unterstützung und Pflege für andere geleistet werden [19], was sich mit dem „Stress-process“-Modell [13] durch dabei entstehende Belastungen erklären lässt.

Die Pandemie macht bestehende Ungleichheiten deutlich und verstärkt diese, auch in diesem Bereich: Auf Basis des Deutschen Alterssurvey zeigt sich für den Zeitraum Juni/Juli 2020 ein Anstieg geleisteter Pflege in der Altersgruppe 40+ im Vergleich zum Jahr 2017. Pflegende Angehörige, und hier v. a. Frauen, berichten seit der Pandemie von schlechterer mentaler Gesundheit und gestiegener Einsamkeit, was für Nicht-Pflegende nicht in diesem Maße gilt [9]. Ähnliches zeigen Befragungen pflegender Angehöriger, die eine nach dem SGB XI pflegebedürftige Person versorgten, aus dem Frühjahr und Sommer 2020. Für ca. ein Drittel hat sich die Pflegesituation verschlechtert, und die subjektive Belastung [2] sowie der Zeitaufwand für Pflege sind gestiegen [16].

Auch zur Situation von unterstützungsbedürftigen Personen gibt es erste Studien. Zu Beginn der Pandemie wurden Pflegekräfte in Pflegeinrichtungen zu den Auswirkungen für die Gepflegten befragt. Demnach führen die für die BewohnerInnen nicht immer nachvollziehbaren Einschränkungen, u. a. des Besuchsrechts, zu mentaler Belastung [7]. In einer Telefonbefragung wurden im Herbst 2020 in Privathaushalten lebende Personen (75 bis 100 Jahre) zu den Pandemiefolgen befragt: Etwa ein Viertel der 500 Befragten berichtete von vielen oder gelegentlichen Momenten der depressiven Verstimmung und Langeweile. Zu einem etwas geringen Anteil gaben die Befragten an, sich mindestens hin und wieder alleingelassen zu fühlen (ca. 15 %). Von denjenigen, die bisher praktische oder pflegerische Hilfe bekamen, gab die Mehrheit aber an, diese Unterstützung weiterhin zu erhalten [8].

Der Forschungsstand zeigt, dass sich private Unterstützung und Wohlbefinden durch die COVID-19-Pandemie verändert haben. Ursächlich sind aufseiten der Unterstützungspersonen vermutlich der gestiegene Betreuungsaufwand und gesunkene Unterstützung durch Dritte [2, 9, 16]. Bei den unterstützungsbedürftigen Personen können es der Verzicht auf gesundheitliche Dienstleistungen sowie der Rückgang der benötigten Unterstützung insgesamt sein [20]. Zudem ist davon auszugehen, dass eine Reduktion der sozialen Kontakte das Wohlbefinden allgemein verringert hat [3].

Die vorliegende Studie erweitert den Forschungsstand zu Unterstützung und Wohlbefinden von Personen im mittleren und im höheren Erwachsenenalter, indem sie das Thema breiter auffasst als die bisherigen Studien, u. a. was die Inhalte (wie z. B. Hilfe im Haushalt, Hilfe bei Schreibarbeiten, sonstige Hilfen) betrifft. Die Datenerhebung fand zudem zu einem frühen Zeitpunkt der Pandemie statt. Ob hier schon Probleme in der Unterstützung älterer Angehöriger entstanden, und inwiefern sich dies auf das subjektive Wohlbefinden auswirkte, wurde bislang nicht untersucht.

## Studiendesign und Methode

In der Pilotstudie „Gesundheit und Unterstützung in Zeiten von Corona“ wurde der Einfluss der Pandemie auf Menschen im Alter von 40+ Jahren in Privathaushalten untersucht. Die Erhebung fand von Mai bis Juli 2020 statt, als langsam Lockerungen der Pandemiemaßnahmen auch in den am stärksten betroffenen Bundesländern eingeführt wurden. So wurden in Nordrhein-Westfalen im Mai die Schulen schrittweise wieder geöffnet, gefolgt von Restaurants. Ab Mitte Juni wurden dann Restriktionen, die private Feste und Freizeiteinrichtungen betrafen, gelockert. In Altenpflegeeinrichtungen galt im Befragungszeitraum kein generelles Besuchsverbot mehr, aber strenge Schutzmaßnahmen für Besuche waren bis Anfang Juli noch in Kraft [10]. Der 7‑Tage-Inzidenzwert, erfasste Neuansteckungen pro 100.000 EinwohnerInnen in den letzten 7 Tagen, betrug zum Monatsanfang Mai, Juni und Juli deutschlandweit 7,2, bzw. 2,6 und 2,9 [15].

Bei der Studie handelt es sich um eine standardisierte quantitative Online-Befragung auf Basis einer Gelegenheitsstichprobe [14]. Sofern gewünscht, konnte der Fragebogen in einem Telefoninterview mit den ProjektmitarbeiterInnen durchgegangen werden. In der Befragung wurden u. a. Änderungen im Erhalt und im Leisten von Unterstützung sowie Probleme in der Betreuung älterer Angehöriger im Zuge der Pandemie erfasst. Subjektives Wohlbefinden wurde mit der Frage nach allgemeiner Lebenszufriedenheit sowie mit der Frage danach, ob man Gesellschaft vermisst, als Einsamkeitsitem [17] erhoben. Die TeilnehmerInnen wurden explizit um einen Vergleich ihrer Situation *vor Ausbreitung* der COVID-19-Pandemie, vor Ende Februar 2020, bevor die ersten Fälle in Deutschland bekannt wurden, mit der Zeit *nach Ausbreitung* der Pandemie ab März 2020 gebeten. Erhoben wurden zudem soziodemografische Merkmale wie Bildung, Alter und Geschlecht.

Im Folgenden werden deskriptive Statistiken zu COVID-19-bedingten Veränderungen erhaltener und geleisteter Unterstützung, Betreuungsproblemen mit älteren Angehörigen sowie Aspekten des subjektiven Wohlbefindens (Lebenszufriedenheit, Einsamkeit), differenziert nach Alter und Geschlecht, präsentiert. Anschließend werden Ergebnisse logistischer Regressionsmodelle gezeigt, die den Zusammenhang zwischen Unterstützung(sproblemen) und Wohlbefinden beleuchten.

## Ergebnisse

Nach Ausschluss von Befragten, die die Befragung vorzeitig beendeten, betrug die Stichprobe 425 Personen. Unter den Befragten sind Frauen mit 72,0 % häufig vertreten. Das Durchschnittsalter beträgt 58,9 Jahre (zwischen 40 und 91 Jahre), wobei die Altersverteilung mit Ausnahme der Hochaltrigen der in der Gesamtbevölkerung weitgehend entspricht. Die Mehrzahl der Befragten (61,8 %) lebt mit der/dem (Ehe‑)PartnerIn gemeinsam in einem Haushalt; 77,5 % haben mindestens ein Kind. Überproportional sind Personen mit einem Hochschulabschluss (53,1 %) in der Stichprobe vertreten [6, 14].

### Unterstützung

Die TeilnehmerInnen wurden gefragt, wie es seit Ausbreitung von COVID-19 um die Möglichkeit, andere zu unterstützen, und um den Erhalt von Unterstützung stand (Tab. 1). Etwa je 20 % gaben an, die benötigte Unterstützung im Alltag (teilweise) nicht erhalten zu haben (Frauen: 11,5 und 9,8 %; Männer: 12,1 und 8,1 %). Etwa 30 % (Frauen: 18,4 und 13,3 %) bzw. 20 % (Männer: 14,3 und 7,1 %) gaben an, dass sie (teilweise) selbst die von Personen aus ihrem sozialen Umfeld benötigte Unterstützung nicht leisten konnten.

**Table 1:** 

	Frauen	Männer
Benötigte Unterstützung erhalten^1^ (*N* = 334)		
Ja	22,6	15,2
Teilweise	11,5	12,1
Nein	9,8	8,1
Trifft nicht zu	56,2	64,7
Benötigte Unterstützung leisten^2^ (*N* = 353)		
Ja	29,4	24,5
Teilweise	18,4	14,3
Nein	13,3	7,1
Trifft nicht zu	38,8	54,1

Daten: „Alles in allem betrachtet: Bitte geben Sie für die folgenden Aussagen die Antwort an, die auf Sie zutrifft. Seit der Ausbreitung des Coronavirus in Deutschland … ^1^ erhalte ich genügend Unterstützung, ^2^ kann ich die nötige Unterstützung leisten“, keine Information zur Intensität, TU Dortmund Pilotstudie, Diff. zur Stichprobe durch fehlende Werte, nicht gewichtet

Unterstützungsmuster unterscheiden sich stark zwischen den Altersgruppen (Tab. 2). So gaben 39,5 % der 40- bis 49-Jährigen an, die benötigte Unterstützung seit der Ausbreitung von COVID-19 (teilweise) nicht erhalten zu haben (40–49 J.: 17,1 und 22,4 %). Umgekehrt lag der Anteil derer, die die benötigte Unterstützung für andere nicht leisten konnten, bis zu einem Alter von 79 Jahren bei rund einem Zehntel der Befragten (9,9 bis 11,3 %). Bei den Befragten im Alter von 80 Jahren und älter sagten 38,5 %, dass sie seit Ausbreitung von COVID-19 die benötigte Unterstützung für andere nicht leisten konnten.

**Table 2:** 

	40–49 J	50–59 J	60–69 J	70–79 J	80+ J
Benötigte Unterstützung erhalten (*N* = 334)					
Ja	15,8	20,9	27,0	7,6	42,9
Teilweise	17,1	13,2	9,0	7,6	7,1
Nein	22,4	4,4	7,0	3,8	7,1
Trifft nicht zu	44,7	61,5	57,0	81,1	42,9
Benötigte Unterstützung leisten (*N* = 353)					
Ja	38,3	34,3	24,0	11,3	15,4
Teilweise	23,5	21,6	11,5	11,3	15,4
Nein	9,9	7,8	13,5	11,3	38,5
Trifft nicht zu	28,4	36,3	51,0	66,0	30,8

Daten: Frage: Tab. 1, TU Dortmund Pilotstudie, Diff. zur Stichprobe durch fehlende Werte, nicht gewichtet

Frauen berichteten etwas häufiger von Problemen in der Betreuung älterer Angehöriger (16,3 % vs. 14,2 %). Nach Alter unterschieden kamen Betreuungsprobleme am häufigsten bei den 50- bis 59-Jährigen (22,4 %) und 60- bis 69-Jährigen (17,8 %) vor (Tab. 3).

**Table 3:** 

(*N* = 371)	Ja/teilweise	Nein	Trifft nicht zu
*Geschlecht*			
Frauen	16,3	33,2	50,6
Männer	14,2	35,9	50,0
*Alter*			
40–49 J	10,7	50,0	39,3
50–59 J	22,4	38,4	39,3
60–69 J	17,8	18,7	63,6
70–79 J	5,7	24,5	69,8
80+ J	13,4	53,3	33,3

Daten: „Alles in allem betrachtet: Bitte geben Sie für die folgenden Aussagen die Antwort an, die auf Sie zutrifft. Seit der Ausbreitung des Coronavirus in Deutschland … ^1^ habe ich ein Betreuungsproblem mit älteren Angehörigen“, TU Dortmund Pilotstudie, Diff. zur Stichprobe durch fehlende Werte, nicht gewichtet

### Wohlbefinden

Die durchschnittliche Lebenszufriedenheit vor Beginn der Pandemie (retrospektiv zum Befragungszeitpunkt eingeschätzt) betrug bei Frauen wie Männern 8 (± 1,6) bzw. 7,9 (± 1,6). Mit der Ausbreitung von COVID-19 berichteten Frauen eine durchschnittlich stärkere beeinträchtigte Lebenszufriedenheit von 6,6 (± 2,1) im Vergleich zu Männern mit 7,0 (± 1,7). Der Altersgruppenvergleich zeigte, dass die 60- bis 69-Jährigen und die 80+ im Zuge der Pandemie besonders starke Einbußen der Lebenszufriedenheit erlebten (Tab. 4).

**Table 4:** 

	Vor COVID-19	Seit COVID-19
40–49 J	7,7 (1,4)	6,8 (1,8) ^a^
50–59 J	7,8 (1,8)	6,8 (1,9) ^a^
60–69 J	8,0 (1,4)	6,2 (2,2) ^a^
70–79 J	8,4 (1,7)	7,3 (1,8) ^a^
80+ J	8,5 (1,7)	6,5 (2,7) ^a^

Daten: „Auf einer Skala von 0 bis 10, bei der 0 ‚voll unzufrieden‘ und 10 ‚voll zufrieden‘ bedeutet, wie zufrieden waren bzw. sind Sie mit Ihrem Leben?“, TU Dortmund Pilotstudie

^a^ Diff. ist signifikant, 5 % Sign.-Niveau, zweiseitiger t‑Test für abhängige Stichproben, Diff. zur Stichprobe durch fehlende Werte, nicht gewichtet

**Figure 1:**
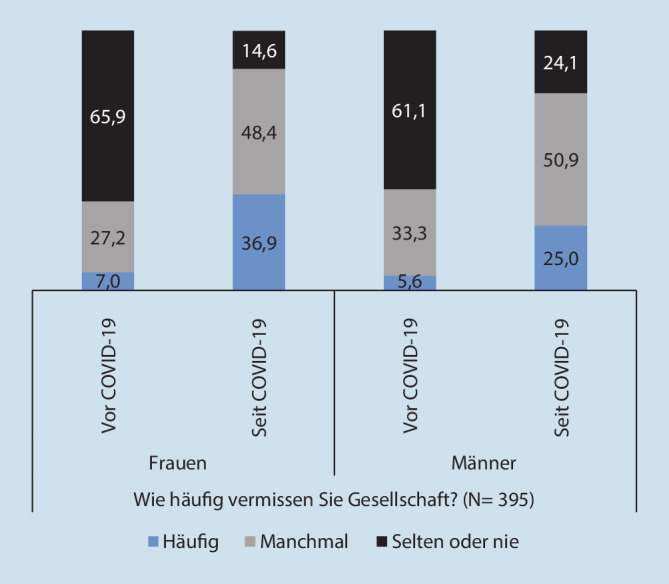

Veränderungen sind, wie erwartet, auch für das Einsamkeitsempfinden (hier dargestellt anhand des Items „Gesellschaft vermissen“) feststellbar (Abb. 1). In der Zeit vor der Ausbreitung von COVID-19 war der Anteil an Personen, die „häufig Gesellschaft vermissten“, mit 7,0 % (Frauen) und 5,6 % (Männer) eher gering. Seit der Ausbreitung des Virus hat das Gefühl von fehlender Gesellschaft drastisch zugenommen. Nun gaben 36,9 % (Frauen) und 25,0 % (Männer) an, sich „häufig“ einsam zu fühlen.

**Figure 2:**
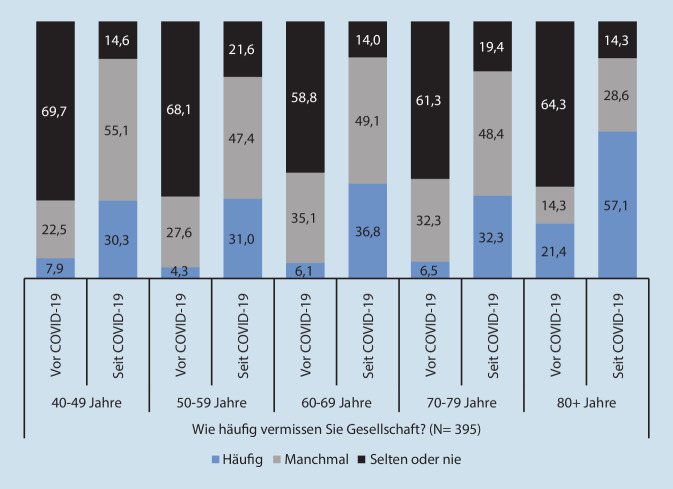

Zudem zeigten sich Altersunterschiede im Einsamkeitsempfinden. Vor COVID-19 lag der Anteil derjenigen, die „häufig Gesellschaft vermissten“, nur in der Altersgruppe 80+ Jahre bei über 20 %. Seit COVID-19 gab in allen Altersgruppen mindestens ein Drittel an, dies häufig zu tun. Die Zunahme von Einsamkeit war in der Altersgruppe 80+ am deutlichsten (Abb. 2). Alle hier berichteten Änderungen sind statistisch signifikant (Wilcoxon-Vorzeichen-Rang-Test).

Insgesamt ergaben sich also im Zuge der Pandemie umfassende Änderungen in Unterstützungsmustern und im subjektiven Wohlbefinden von Frauen und Männern sowohl mittleren als auch höheren Alters.

### Multivariate Ergebnisse

Aus der COVID-19-Forschung ist bisher bekannt, dass sich das Wohlbefinden durch Kontaktbeschränkungen [3, 7, 8] und veränderte Pflegebedingungen [9, 16] verringern kann. Davon ausgehend untersuchen die folgenden Analysen den Zusammenhang zwischen veränderten Unterstützungsmustern und Betreuungsproblemen mit dem subjektiven Wohlbefinden der Befragten.

Als abhängige Variablen dienen zwei Indikatoren des Wohlbefindens: die Veränderungen – vor und seit COVID-19 – der Lebenszufriedenheit und des Gefühls, häufig die Gesellschaft anderer zu vermissen. In beiden Fällen wurden logistische Regressionsmodelle geschätzt, in denen jeweils eine unabhängige Variable zu Unterstützungsmustern enthalten ist (Tab. 5). Die Referenz ist die Antwortmöglichkeit „trifft nicht zu“, d. h., dass diese Personen keine Unterstützung benötigten (M1), keine Unterstützung leisteten (M2) oder keine älteren Angehörige (M3) betreuten.

**Table 5:** 

	Geringere Lebenszufriedenheit		Häufiger „Gesellschaft vermissen“	
	AME	SF	AME	SF
M1: Benötigte Unterstützung erhalten				
–	M1.1		M1.2	
*Ref. Trifft nicht zu*				
Ja	0,029	0,077	0,064	0,076
Nein/teilweise	0,096	0,077	0,081	0,077
M2: Benötigte Unterstützung leisten				
*–*	M2.1		M2.2	
*Ref. Trifft nicht zu*				
Ja	−0,047	0,076	−0,170*	0,075
Nein/teilweise	0,052	0,074	0,009	0,072
M3: Betreuungsprobleme mit älteren Angehörigen				
–	M3.1		M3.2	
*Ref. Trifft nicht zu*				
Ja/teilweise	−0,011	0,095	0,174*	0,085
Nein	0,000	0,069	−0,014	0,070

Daten: TU Dortmund Pilotstudie,* n* = *275*

*AME „*average marginal effect“,* SF* Standardfehler

Sign.-Niveau: *p *<* 0,05, **p *<* 0,01, ***p *<* *0,001; *Kontrolle: Geschlecht, Alter (60+ vs. < 60), Kinder (mindestens ein Kind vs. kein Kind), Familienstand (verheiratet/PartnerIn vs. nicht verheiratet/kein/e PartnerIn), Bildung (Hochschulbildung vs. niedrigere Bildung), Monat der Befragung, nicht gewichtet

Die Wahrscheinlichkeit, häufiger Gesellschaft zu vermissen, war für Personen, die benötigte Unterstützung leisten konnten, geringer als für Personen, die keine Unterstützung leisteten (M2.2, b = −0,170). Betreuungsprobleme mit älteren Angehörigen gingen, verglichen mit Personen, die keine älteren Angehörigen betreuten, mit einer höheren Wahrscheinlichkeit einher, Gesellschaft häufiger zu vermissen (M3.2, b = 0,174).

## Diskussion

Die Ergebnisse der Pilotstudie zeigen im Einklang mit den Erwartungen deutliche Veränderungen in Unterstützungsmustern und Wohlbefinden im Frühsommer der Pandemie. Ein beachtlicher Teil der Befragten gab an, nicht mehr ausreichend unterstützt zu werden oder andere nicht mehr ausreichend unterstützen zu können. Insbesondere Personen im höheren Alter zogen sich aus der Unterstützung für andere zurück, v. a. aus (gegenseitiger) Angst vor Ansteckung [5, 6].

Die Auswirkungen der Pandemie und ihrer Begleiterscheinungen auf das subjektive Wohlbefinden zeigten sich ebenfalls deutlich. Viele Befragte berichteten von einem verringerten Wohlbefinden. Die stärksten Veränderungen sind bei Frauen und Hochaltrigen festzustellen, die auch vor der Pandemie schon ein erhöhtes Einsamkeitsrisiko trugen [11, 12]. Die Befragung legt nahe, dass sich solche Ungleichheiten im Zuge der Pandemie weiter verschärft haben.

So überrascht die stärkere Abnahme des Wohlbefindens bei Frauen nicht, die einen Großteil der in der Pandemie erschwerten Sorgearbeit für ältere Angehörige leisten [9, 16]. Die Analysen zeigen, dass Probleme bei der Unterstützung für ältere Angehörige mit vermehrter Einsamkeit verbunden sind. Das lässt sich so interpretieren, dass die Reduktion z. B. professioneller Unterstützung durch Dritte mehr Verantwortung für Sorgende bedeutete, die mit dem Gefühl einherging, auf sich alleine gestellt zu sein. Bestehen solche Herausforderungen nicht, scheinen Unterstützungsleistungen an andere, vermutlich aufgrund des vermehrten Kontakts und der Sinnerfüllung, mit geringerer Einsamkeit verbunden zu sein.

Einordnend muss gesagt werden, dass in Frauen und Hochgebildete in der Pilotstudie überrepräsentiert sind, während Hochaltrige unterrepräsentiert sind. Auch unter Kontrolle von Geschlecht, Bildung und Alter lässt sich vermuten, dass die Ergebnisse als „konservative“ Schätzung im Hinblick auf soziale und mentale Auswirkungen nur die Spitze des Eisbergs in einer Gruppe vergleichsweise privilegierter Personen zeigen. Auch der retrospektive Charakter mancher Fragen birgt die Gefahr von Verzerrungen. Insgesamt stehen die Ergebnisse aber im Einklang mit bisherigen Studien, die verringertes Wohlbefinden, gestiegene Belastungen und verstärkte Einsamkeit bei Älteren auch im Zusammenhang mit der Angehörigenpflege im Zuge der Pandemie nachweisen. Die vorgelegten Analysen erweitern den Forschungsstand, indem sie den Zusammenhang von veränderten Unterstützungsmustern und dem Wohlbefinden näher untersuchen. Eine weitere Besonderheit der Pilotstudie liegt im frühen Erhebungszeitraum in der Anfangsphase der Pandemie, in der drastische Maßnahmen zur Eindämmung des Virus eingeleitet worden sind.

Es bleibt abzuwarten, wie sich Unterstützung und Wohlbefinden weiter verändern – auch vor dem Hintergrund erneut beschlossener Kontaktbeschränkungen. Diese Studie gibt Hinweise darauf, welche Gruppen ein besonderes Risiko für mentale und soziale Belastungen tragen. Zukünftige Analysen auf Basis mittlerweile vorliegender repräsentativer Befragungen (z. B. „Sozio-ökonomische Faktoren und Folgen der Verbreitung des Coronavirus in Deutschland“, SOEP-CoV) können daran anknüpfen. Wichtige Forschungsfragen betreffen z. B. die Rolle von Kommunikationstechnologien zur Verringerung von Einsamkeit sowie zur (sozialen und kulturellen) Partizipation Älterer. Spannend sind zudem Fragen der Veränderung von Altersbildern und intergenerationaler Solidarität (u. a. Enkelbetreuung) durch die Pandemie.

## Fazit für die Praxis

- Mit COVID-19 gehen Veränderungen im Unterstützungsverhalten einher, die die Gefahr einer unzureichenden Versorgung bergen.
- Die Maßnahmen zur Eindämmung der Pandemie reduzieren die Lebenszufriedenheit und erhöhen das Einsamkeitsempfinden.
- Kontaktbeschränkungen erschweren u. a. den Austausch notweniger Unterstützung und sollten mit Bedacht eingesetzt werden.
